# A Sphingosine-1-Phosphate Receptor Modulator Attenuated Secondary Brain Injury and Improved Neurological Functions of Mice after ICH

**DOI:** 10.1155/2020/3214350

**Published:** 2020-09-04

**Authors:** T. Bobinger, T. Bäuerle, L. Seyler, S. v Horsten, S. Schwab, H. B. Huttner, A. Manaenko

**Affiliations:** ^1^Department of Neurology of the Friedrich-Alexander-University Erlangen-Nuremberg, Germany; ^2^Department of Radiology of the Friedrich-Alexander-University Erlangen-Nuremberg, Germany; ^3^Department of Experimental Therapy, University Hospital Erlangen, and Preclinical Experimental Animal Center, Friedrich-Alexander-University Erlangen-Nuremberg, Germany

## Abstract

**Background:**

Stroke activates the immune system and induces brain infiltration by immune cells, aggravating brain injury. Poststroke immunomodulation via (S1P-)receptor modulation is beneficial; however, the S1P-modulator in clinical use (FTY-720) is unspecific, and undesirable side effects have been reported. Previously, we tested effects of a novel selective S1P-receptor modulator, Siponimod, on ICH-induced brain injury in acute stage of the disease. In the current study, we investigated whether protective effects of Siponimod, evaluated in a short-term study, will protect the brain of ICH animals at long term as well.

**Methods:**

134 C57BL/6N mice were divided into sham and ICH-operated groups. Collagenase model of ICH was employed. ICH animals were divided into Siponimod treated and nontreated. Dose- and time-dependent effects of Siponimod were investigated. Contraplay between development of brain injury and the number of lymphocytes infiltrating the brain was investigated by forelimb placing, T-Maze test, brain water content calculation, MRI scanning, and immunostaining.

**Results:**

Depending on the therapeutic strategy, Siponimod attenuated the development of brain edema, decreased ICH-induced ventriculomegaly and improved neurological functions of animals after ICH. It was associated with less lymphocytes in the brain of ICH animals.

**Conclusion:**

Siponimod is able to decrease the brain injury and improves neurological functions of animals after ICH.

## 1. Introduction

The immune system decisively participates on the development of secondary brain injury after stroke. In patients, stroke is associated with an increased number of peripheral inflammation markers (white blood cell (WBC) and peripheral neutrophil count). In animal stroke models, the poststroke brain infiltration by splenocytes significantly contributes to the inflammation and development of secondary brain injury after stroke, resulting in the development of perihematomal brain edema in the acute stage and ventriculomegaly as well as brain atrophy in the chronical stage of disease [1–6].

Previously, beneficial effects of a clinically approved sphingosine-1-phosphate receptor (S1PR) modulator, FTY-720, were demonstrated in preclinical and clinical settings [1, 7, 8]. However, FTY-720 is an unselective modulator of S1PR and side effects are reported [9]. Furthermore, stroke induced immunosuppression, and the infection is a leading cause of death of stroke patients [9]. The long half-life time of FTY-720 might be an explanation for FTY-720-induced lethal infection which is not desirable [10]. Compared to FTY-720, Siponimod has significant lower half-life time and acts only on two out of five receptor isoforms, which are mostly expressed on immune cells [11, 12]. That should be able to avoid the undesirable side effects of the immunomodulation.

In our proceeded paper (https://www.ncbi.nlm.nih.gov/pubmed/31558140), we demonstrated that a second-generation selective S1P-receptor modulator, Siponimod, dose-dependently attenuated the development of secondary brain injury in acute stage of ICH, resulting in improved neurological functions of ICH animals in a short-time study [13]. Furthermore, we demonstrated that in long term, Siponimod increased the survival rate of treated compared to nontreated animals. However, the long-term effects of Siponimod have not been further evaluated yet [13].

In the current study, we investigated whether beneficial Siponimod-induced short-term effects will also induce a long-term brain protection, resulting in an attenuation of post-ICH ventriculomegaly and brain atrophy as well as neurological dysfunctions.

## 2. Methods and Materials

All experiments were conducted with the approval of the Government of Unterfranken (approval number 55.2-2532-2-206).

### 2.1. Intracerebral Hemorrhage Mouse Model

The collagenase model of ICH on mice was used [13]. Mice were anesthetized with ketamine (100 mg/kg) and xylazine (10 mg/kg, intraperitoneal (i.p.) injection), then positioned prone in a stereotactic head frame. Animal core temperature (37°C) was maintained by a thermostat-controlled warming blanket. The midline scalp was incised from the nasion to the superior nuchal line. The calvarium was exposed. Using a speed drill device (Fine Scientific Tools, Foster City, CA), a 1 mm burr hole 0.9 mm posterior to the bregma and 2.2 mm to the right of the midline was prepared. A 26 G needle on a Hamilton syringe 3.5 mm was induced into the right deep cortex/basal ganglia at a rate of 1 mm/minute. Collagenase solution (0.075 U in 0.5 *μ*L saline, VII-S; Sigma, St. Louis, MO) was infused at a rate of 0.25 *μ*L/minute using an infusion pump (Stoelting, Wood Dale, IL). After 10 minutes, the needle was withdrawn, the incision was closed, and the mice were allowed to recover. Sham-operated animals received a needle trauma only.

### 2.2. Treatment and Experimental Groups

134 C57BL/6N mice (10 weeks, 20-24 g) were obtained from Charles River (Sulzfeld, Germany). Mice were housed in a room with constant temperature (25°C), humidity control, and a 12/12 h light/dark cycle, free access to food and water.

0.3 and 3.0 (mg/kg of body weight) of Siponimod in 0.5% DMSO (intraperitoneally) were tested. We used either a single (30-minute post-ICH) or multiple (three times: 30 minutes, 24 and 48 hours post-ICH) Siponimod administration.

In the short-time study, animals were neurologically tested 24 and 72 hours after ICH. After the final testing, animals were euthanized and the development of brain edema was investigated by brain water content calculation using the wet/dry method.

In the long-term study, spatial learning was evaluated by a T-Maze test. The progress of brain atrophy and ventriculomegaly was investigated by MRI.

### 2.3. Evaluation of Brain Edema Development

The development of brain edema was investigated by calculating brain water content [14]. The dry/wet method was used. Briefly, mice were euthanized under deep anesthesia. Brains were removed and divided into five parts: the ipsilateral and contralateral basal ganglia, the ipsilateral and contralateral cortex, and the cerebellum. The cerebellum was used as an internal control for brain water content. Tissue samples were weighed on an electronic analytical balance to the nearest 0.1 mg to obtain the wet weight. The tissue was then dried at 100°C for 48 h to determine the dry weight. Brain water content (%) was calculated as [(wet weight − dry weight)/wet weight]∗100.

### 2.4. Neurobehavioral Function Test

Neurological functions were assessed by forelimb placement (short term, motor functions) and T-Maze (long term, spatial functions) tests [15, 16].

For the limb placement test, the animals were held by their trunk positioned parallel to a table top and slowly moved up and down, allowing the vibrissae on one side of the head to brush along the table surface. Refractory placements of the impaired (left) forelimb were evaluated, and a score was calculated as the number of successful forelimb placements out of 10 consecutive trials [15].

The T-Maze assessed short-term (working) memory [17]. Briefly, mice were placed into the stem of a maze and allowed to explore until one arm was chosen. From the sequence of 10 trials, of left and right arm choices, the rate of spontaneous alternation was calculated (range 0% (no alteration/trial) to 100% (complete, alternations/trial)) [16].

### 2.5. Tissue Processing and Immunostaining

Under final anesthesia, animals were perfused via intracardiac puncture with ice cold PBS followed by perfusion with 10% PFA. Brain samples were stored in PFA overnight. They were then cryopreserved with increasing (10, 20, and 30%) concentration of sucrose. Staining was conducted according to vendor recommendation.

Positive cells were quantified in the perihematomal region (*N* = 3 per group) by an experimenter blinded to the experimental procedure.

### 2.6. MRI Experiments

MRI was performed on a 7 Tesla scanner dedicated for small animal examinations using a brain coil (ClinScan, Bruker, Ettlingen, Germany). Under isoflurane anesthesia, mice were imaged with the following sequence: 3D mprage (voxel size: 0, 117 × 0, 117 × 0, 25; TR: 2500 ms; TE: 2.82 ms; TA: 22 min). The volumes of ventricles were quantified on MR images using postprocessed Chimaera's segmentation tool (Chimaera GmbH, Erlangen, Germany).

### 2.7. Statistics

Analysis was performed using GraphPad Prism 7.00 software. The data distribution was tested by the D'Agostino and Pearson normality test, and all the data met the criteria of normal distribution. Statistical differences were analyzed with one-way analysis of variance (ANOVA) followed by Dunnett's multiple comparisons test. Statistical significance was defined as *P* < 0.05. Data are expressed as mean ± standard error of mean.

## 3. Results

### 3.1. Siponimod Attenuated the Development of ICH-Induced Brain Edema

**Figure 1:**
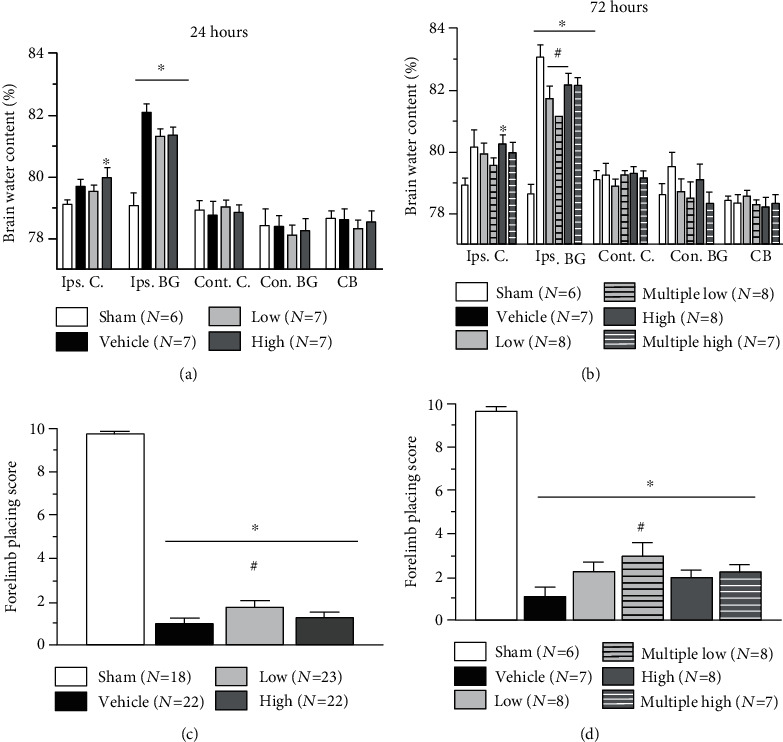
Effect of Siponimod on the development of brain edema and neurological dysfunctions after ICH. Compared to sham-operated animals, collagenase-induced ICH resulted in increase in brain water content both at 24 ∗(a) and 72 (b) hours after insult. Development of brain edema was associated with neurological dysfunctions as evaluated through the forelimb placing test at 24 and 72 hours after ICH (c, d). Although at 24 hours after ICH Siponimod treatment showed only tendency to decrease post-ICH brain water content, treatment with low concentration of the drug significantly attenuated development of neurological dysfunctions after ICH as evaluated using the forelimb placing test (a and c, respectively). At 72 hours, treatment with low concentration of Siponimod resulted in significant decrease in post-ICH brain water content, whereby the multiple treatment improved neurological functions of ICH animals significantly (b and d, respectively). (∗*P* < 0.05 vs. sham, ^#^*P* < 0.05 vs. vehicle). Values are expressed as mean ± SD.

Compared to sham-operated animals, collagenase-induced ICH resulted in significant increase of brain water content in the basal ganglia of all ICH animals evaluated at both 24 and 72 hours after ICH. An increase in brain water content was observed at the ipsilateral cortex as well (Figures 1(a) and 1(b)).

Treatment with both high and low concentrations of Siponimod showed clear tendency for the decrease in post-ICH brain water content in the basal ganglia at 24 hours. The tendency, however, did not reach statistical significance (Figure 1(a)).

Furthermore, both single and multiple administrations of Siponimod low dose significantly decrease post-ICH brain water content in the basal ganglia at 72 hours. Multiple administration of Siponimod low dose resulted in the reduction of brain water content in the ipsilateral basal ganglia as well. The difference between treated and nontreated animals, however, did not reach statistical significance (Figure 1(b)).

### 3.2. Siponimod Improved the Neurological Motor Functions of Animals after ICH

All ICH animals demonstrated significant neurological dysfunctions evaluated by limb placing test 24 and 72 hours after impact (Figures 1(c) and 1(d)). Administration of a low but not a high dose of Siponimod improved neurological functions of ICH at 24 hours after ICH (Figure 1(c)). At 72 hours, ICH animals treated multiple times with low dose of ICH demonstrated significantly better performance at a limb placement test, as compared to nontreated animals (Figure 1(d)).

### 3.3. Siponimod Attenuated ICH-Induced Brain Injury 10 Days after ICH

**Figure 2:**
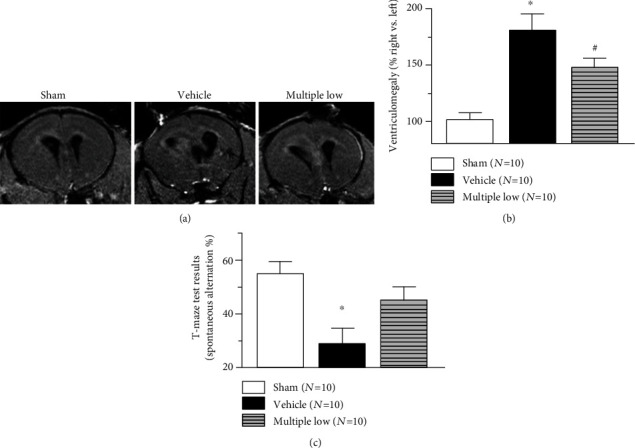
Effect of Siponimod on ventriculomegaly and cognitive functions after ICH. Representative MRI scans in corresponding locations through the ventricles (coronal orientation). Significant increase of ipsilateral ventricle size was observed 10 days after ICH. Multiple treatment with Siponimod attenuated the development of post-ICH ventriculomegaly (b). Post-ICH ventriculomegaly was associated with impairment of spatial functions (working memory), which resulted in the decrease of spontaneous alteration in the T-Maze test. Multiple treatment increased the number of spontaneous alterations, indicating that Siponimod improved working memory of ICH animals (c). (∗*P* < 0.05 vs. sham, ^#^*P* < 0.05 vs. vehicle). Values are expressed as mean ± SD.

A significant increase in the right ventricle's size was observed 10 days post-ICH. Furthermore, we demonstrated that multiple administration of low dose of Siponimod attenuated the development of ICH-induced ventriculomegaly (Figure 2(b)).

### 3.4. Siponimod Improved the Cognitive Functions of Animals after ICH

For evaluation of cognitive functions of animals, a T-Maze test (a test evaluating impairment of working memory) was used. ICH resulted in significant decrease of spontaneous alteration rate. Multiple treatments with low dose of Siponimod reverse this effect. The difference between sham-operated and ICH animals with multiple treatments with Siponimod was not statistical significant.

### 3.5. Siponimod Attenuated Brain Infiltration by Systemic Immune Cells

**Figure 3:**
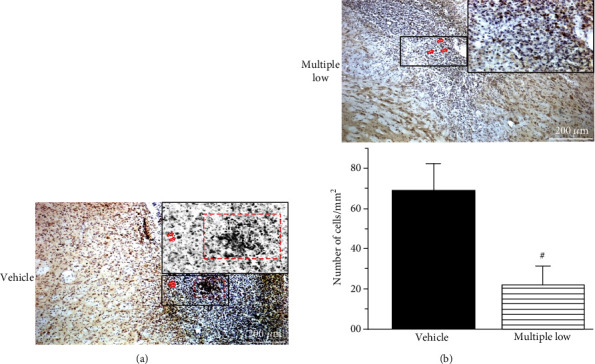
At day 10, Siponimod decreased the number of CD3-positive cells in the brain after ICH ((a) untreated vs. (b) treated ICH animals).(c) Quantification of the results (^#^*P* < 0.05 vs. vehicle). Values are expressed as mean ± SD.

At day 10 post-ICH, Siponimod decreased the number of CD3 but not CD19-positive cells in the perihematomal area of treated animals (Figure 3(b)) compared to nontreated animals (Figure 3(a)).

## 4. Discussion

In this study, we continued our investigation on the effects of a clinically approved S1P-receptor modulator, Siponimod. We investigated the effects of Siponimod on the development of both short- and long-term secondary brain injuries and neurological deficits after ICH in mice.

Previously, it has been demonstrated that the first-generation S1P-receptor modulator, FTY-720, attenuated post-ICH brain injury in mice [1]. However, FTY-720 is unselective, and its side effects (bradycardia or hypertension) and the occurrence of fatal infection, probably due to a long half-life time, were reported [10, 18]. Siponimod is an agonist of only two isoforms S1P-R1 and S1P-R5 and has a short half-life time [15].

Effects of Siponimod on the ICH-induced brain damage have not been investigated yet. In the first part of the study, we investigated the effects of Siponimod on the development of brain edema. We demonstrated that, in agreement with previous reports, collagenase-induced bleeding results in the disruption of BBB and consequently in the development of posthemorrhagic brain edema. For investigation of brain edema development, we used in this study the “gold standard,” the calculation of brain water content *via* the “wet/dry” method [14, 15, 19]. At 24 hours after ICH induction, treatment with both low and high concentrations showed a tendency of decreasing hemorrhage-induced brain water content elevation in the ipsilateral basal ganglia. The treatment had no effect on brain water content in the ipsilateral cortex; on the contrary, the difference in brain water content between sham and ICH animals, treated in high concentration, remained statistically significant in this brain region. In agreement with this observation, we were able to demonstrate that high concentration of Siponimod did not improve neurological functions of animals. The low-concentration treatment attenuated the impairment of neurological functions.

72 hours after ICH, the effects of one *vs.* multiple-injections of both low and high concentrations of the Siponimod have been compared. We confirmed the results of our 24-hour study and demonstrated that low concentration significantly attenuated water content in the basal ganglia of treated animals compared with nontreated animals. The multiple administration of low Siponimod concentration resulted in clear but only a tendency of decrease in brain water content in the ipsilateral cortex and the contralateral basal ganglia as well. The effect of drug on brain water content was associated with a significantly improved performance of animals on the forelimb placing test.

The high concentration of the drug was ineffective regardless whether it was administrated in single or multiple mode.

It has been demonstrated previously that while systemic administration of the Siponimod induced lymphopenia, the direct intracerebral administration of the drug had almost no effect on peripheral lymphocyte count [20]. We hypothesize therefore that despite its ability to penetrate BBB, the low concentration of the drug given intraperitoneally affected mostly the peripheral expressed receptors, which control lymphocyte egress from secondary lymphoid organs. There are mounting evidences that stroke-induced increase of the lymphocyte number in circulation results in increased number of lymphocytes infiltrating the brain. This significantly contributes to the rise in inflammation after stroke, and at short term, this results in the development of brain edema [21, 22]. That is in line with our previous observation that the smaller number of cells in blood was accompanied with less brain edema in treated *vs.* nontreated animals.

It is worth to mention that in addition to its effect on lymphocyte egress, S1P-receptors are involved in the maintenance of the BBB functions, and its signaling regulates endothelial tight junction integrity and is able to protect the brain [23–25]. One can assume that activation of the pathway by natural ligands leads to BBB preservation and brain protection after ICH [26]. The inhibition of the pathway induced by high but not low dose of Siponimod might induce BBB dysfunction and damaged brain cells. The detrimental effects overcome beneficial effects of the inhibition of lymphocyte egress. This hypothesis needs to be further investigated.

Next, we proved whether short-term Siponimod-induced production will also benefit long-term outcomes. The time-dependent effect of ICH on the development of ventriculomegaly after collagenase induced ICH on rats was investigated before [3]. Authors observed the increase in ventricular size as early as three days after ICH with the further increase in ventricular size till day 14 [3]. Also, others observed significant increase in ventricular size after collagenase induced ICH [27, 28]. In agreement with these findings, we demonstrated that ICH resulted in the development of ventriculomegaly. The difference in the ventricular size of ICH between nontreated and sham-operated animals was statistically significant. At this part of the project, we only tested the most effective drug concentration and most effective therapeutic strategy, and we demonstrated that multiple administration of low-dose Siponimod resulted in the attenuation of ICH-induced ventriculomegaly.

Pathology of post-ICH ventriculomegaly is not quite clear. ICH-induced inflammation increases permeability of BBB, which allows macromolecules and water to accumulate in an enlarged extracellular space [29]. Furthermore, inflammation induces the cell deaths which leads to the building of cavity around the lesion in the long term [30]. A clinical study demonstrated that there is an association between inflammation and development of hydrocephalus [31]. Even though the pathology of this event needs to be investigated, inflammation seems to play a key role in it as well. It is feasible that attenuation of inflammation and development of brain edema, induced by Siponimod in a short-term study, resulted in long-term brain protection and the decrease in ventricular size.

It has been demonstrated previously that ventriculomegaly is associated with impairment of memory [32, 33]. Inhibition of ventriculomegaly was associated with improvement of memory [34]. We investigated effects of ICH and Siponimod treatment on short-term, working memory by using a short-term (working) memory test, the T-Maze test [17]. Well, in agreement with others, we demonstrated that the significant ICH-induced increase in ventricular size was associated with a significant decrease of spontaneous alteration in the T-Maze. This indicated that in animals with pathological increase of ventricle size, the working memory is impaired. The multiple administration of low-dose Siponimod not only attenuated the development of post-ICH ventriculomegaly but also improved working memory of ICH animals. The difference in the number of spontaneous alterations between sham-operated animals and ICH animals treated with Siponimod was not significant.

Finally, we demonstrated that at day 10, less lymphocytes were present in the brain of animals treated with Siponimod compared with animals which did not receive the treatment. This finding is in agreement with our previous study which demonstrated that the Siponimod treatment significantly decreased the number of circulating lymphocytes in blood of treated compared to nontreated ICH animals [13].

Previously striking increase of inflammatory cytokines and chemokines was demonstrated at 6 and 22 hours after stroke [35]. At these time points, drastic increase in brain water content was also seen [36]. 96 hours after stroke, less inflammation was observed, and at this time point, spleen shrinking, profound (ca. 90%) reduction in the spleen cell number, and apoptosis of spleen cells were detected [37].

The stroke-induced immunosuppression seems to be a result of spleen overactivation. The spleen releases cells in the circulation at the early time point [37]. The cells penetrate the blood brain barrier and contribute to the post-ICH inflammation and development of brain edema. At the time point at which brain edema peaks, the spleen is unable to release more splenocytes in the circulation. This, however, cannot stop the released splenocytes from penetrating BBB and from contributing to post-ICH inflammation.

On the contrary, our treatment intervened at an early time point. We demonstrated that administration of Siponimod as early as 30 minutes after ICH significantly decreases the number of circulating splenocytes which results in the downregulation of the splenocyte number in the brain as well. Due to their decreased number, splenocytes are unable to significantly contribute to post-ICH inflammation. This results in less brain edema. Therefore, the Siponimod-induced immunomodulation at an early time point is beneficial, in contrast to ICH-induced immunosuppression, which can be observed in the delayed time point.

The study has some limitations. Although the pathways underlying S1P modulation have partly been evaluated before [1, 38], the detailed investigation on the molecular level is needed and planned for the next study. Furthermore, the investigation, whether Siponimod would be effective when administered at a later time point and whether Siponimod-induced protection will remain for chronic stage of ICH (one month), will improve our understanding of the Siponimod mode of action.

In summary, a selective, clinically approved, S1P-receptor modulator, Siponimod, is able to attenuate development of brain injury and neurological dysfunction after ICH. This effect was observed both in short- and in long-term studies.

## Data Availability

The data used to support the findings of this study are available from the corresponding author upon request.
